# Attention Mechanism-Based Feature Fusion and Degradation State Classification for Rolling Bearing Performance Assessment

**DOI:** 10.3390/s25164951

**Published:** 2025-08-10

**Authors:** Teng Zhan, Wentao Chen, Congchang Xu, Luoxing Li, Xiaoxi Ding

**Affiliations:** 1 State Key Laboratory of Advanced Design and Manufacturing Technology for Vehicle, Changsha 410082, China; zhteng2021@hnu.edu.cn (T.Z.); xuccae86@hnu.edu.cn (C.X.); llxly2000@hnu.edu.cn (L.L.); 2 Hunan Lince Rolling Stock Equipment Co., Ltd., Zhuzhou 412001, China; wtchen@lince.com.cn; 3 State Key Laboratory of Mechanical Transmission for Advanced Equipment, Chongqing University, Chongqing 400030, China

**Keywords:** rolling bearing, performance degradation assessment, attention mechanism, feature fusion

## Abstract

Rolling bearing failure poses significant risks to mechanical system integrity, potentially leading to catastrophic safety incidents. Current challenges in performance degradation assessment include complex structural characteristics, suboptimal feature selection, and inadequate health index characterization. This study proposes a novel attention mechanism-based feature fusion method for accurate bearing performance assessment. First, we construct a multidimensional feature set encompassing time domain, frequency domain, and time–frequency domain characteristics. A two-stage sensitive feature selection strategy is developed, combining intersection-based primary selection with clustering-based re-selection to eliminate redundancy while preserving correlation, monotonicity, and robustness. Subsequently, an attention mechanism-driven fusion model adaptively weights selected features to generate high-performance health indicators. Experimental validation demonstrates the proposed method’s superiority in degradation characterization through two case studies. The intersection clustering strategy achieves 32% redundancy reduction compared to conventional methods, while the attention-based fusion improves health indicator consistency by 18.7% over principal component analysis. This approach provides an effective solution for equipment health monitoring and early fault warning in industrial applications.

## 1. Introduction

With the improvement of scientific technology and manufacturing level, an increasing number of complex mechanical equipment plays an indispensable role in industrial production and daily life. During the operation of mechanical equipment, mechanical failures of varying degrees will inevitably occur. Leaving equipment to continue operating without the timely detection of equipment failures may lead to serious accidents, which often cause incalculable human and property losses [1]. In order to achieve the predictive maintenance and autonomous protection of equipment, scholars have proposed prediction and health management (PHM) technology [2]. PHM technology firstly uses sensors to obtain equipment operation data, and then promptly discovers possible damage faults of equipment through data analysis on the one hand, and establishes a condition assessment model to effectively judge the status of equipment through monitoring data on the other hand [3]. As one of the most important parts in mechanical transmission system, bearings have a high probability of failure and will seriously affect the operation of the whole machine. It is of great significance for the mechanical equipment safe operation to conduct research on the sensitive feature extraction of rolling bearing degradation, the construction of health index, the construction of state evaluation model and the online monitoring of running state [4].

However, a single feature contains limited information about the bearing condition, so that using a single feature as a health indicator fails to fully reflect the bearing degradation state. With the development of signal analysis techniques, a large number of features have been constructed to describe the degradation state of the equipment, facilitating a more complete characterization of the equipment condition information from multiple aspects. Nevertheless, not all the constructed features are applicable to the remaining bearing life assessment; moreover, too many features may cause data redundancy and increase the computational effort. Therefore, it is valuable to select a subset of features from the high-dimensional feature set that are sensitive to the bearing degradation state and have a small feature dimensionality to reduce the model complexity and computation time and improve the remaining life prediction accuracy [5,6]. Many scholars have conducted in-depth investigations on the selection of sensitive features for rolling bearing condition characterization. Zhang et al. [7] proposed correlation, monotonicity, and robustness evaluation indexes to select some of the features suitable for remaining life prediction, and the best features were selected by weighting the sum of the three evaluation indexes. Yang et al. [8] used the Fisher ratio to select the key sensitive features from many features and discarded the noisy ones. The Fisher ratio method measures the goodness of features by the ratio of intra-group scatter and intra-group spread, and a larger ratio means better features. Geramifard et al. [9] first selected 8 key features from 16 initial features using a genetic algorithm, and then selected the final 3 features from the selected 8 features by taking a correlation measure.

Multidimensional sensitive features fused to obtain health indicators consistent with the degradation state of the equipment are also important for remaining life prediction, and constructed health indicators have an essential impact on the accuracy of remaining life prediction [10]. Principal component analysis (PCA) is one of the effective ways of feature reduction and fusion [11]. Cheng et al. [12] considered that the traditional linear approach of PCA may be limited without considering the nonlinear characteristics of conditional features, and obtained health metrics by fusing multidimensional raw features using a local linear embedding technique. Rai et al. [13] mapped multidimensional features to obtain bearing performance degradation metrics based on a self-organizing mapper, which is a supervised learning model. Y. Shang et al. [14] utilizes a convolutional neural network (CNN) to learn the spatial features from the bearing condition monitoring data, and then employs a stack of bidirectional gate recurrent units (BGRUs) to extract the temporal degrading trend from the data for a more accurate remaining useful life (RUL) prediction.

In summary, most of the current sensitive feature extraction methods of rolling bearing degradation states are based on multi-feature extraction. The fused features can describe the running state of the bearing more comprehensively. However, the content of the above review shows that there are still some problems in the index construction process, such as feature insensitivity, incomplete feature evaluation and unsatisfactory feature fusion. Meanwhile, rolling bearings operate throughout their life through normal, fault, and failure processes. This process is important to determine the starting point of failure and therefore the bearing degradation state for bearing equipment health management and remaining life prediction. Cheng et al. [15] proposed an unsupervised learning kernel spectrum clustering model capable of mapping the input data to a high-dimensional feature space to achieve adaptive sensing of bearing degradation characteristics, and finally achieved the real-time online abnormality identification of rolling bearings. Xia et al. [16] proposed a two-stage remaining life prediction method for rolling bearings using deep learning networks. Firstly, the acquired bearing signals were divided into multiple degradation stages by a deep neural network model based on denoising autoencoder, and then a deep back propagation (BP) neural network was established to achieve the remaining life prediction for each degradation stage. Zhang et al. [17] firstly extracted the features with high correlation with the root mean square value, and then smoothed the features by using the improved Weibull distribution, and finally, the smoothed features were used as the input of the plain Bayesian model to classify the bearing states. Liu et al. [18] proposed a degradation state classification method based on EWMA control charts to achieve a multi-segment segmentation of the bearing degradation states and adaptively switch the stochastic degradation model. Zhao et al. [19] divided the whole life cycle of rolling bearings into three states—the normal state in the initial stable stage, labeled the normal state, which exhibits abnormal behavior; the fault state; and the quasi-fault state between the two—and the grayscale image obtained after wavelet analysis of the vibration signal is learned by using deep convolutional neural networks, and finally, the trained model is used to realize the partitioning of the whole life cycle state of the bearing. Cui et al. [20] established an effective kinetic model of the transient vibration behavior of rolling bearings and obtained a large amount of performance degradation simulation data. A new similarity-based performance degradation dictionary was constructed for RUL prediction, and the degradation process was divided into stable, defect-emerging, defect-expanding, and damage stages. Zhang et al. [21] implemented the proposed waveform entropy index and other conventional features into the long short-term memory neural network to classify the bearing fault states, and also used the optimization-seeking capability of the particle swarm optimization algorithm to optimize the hyperparameters of the network model.

Many scholars have carried out relevant research on the evaluation of rolling bearing performance degradation and the fusion of attention mechanism features. The limitation of single vibration features containing insufficient information and the redundancy in high-dimensional feature sets can be addressed by employing adaptive sensitive feature selection methods to identify degradation-sensitive characteristics [22]. Scholars have explored fusion strategies for heterogeneous features, such as time domain and frequency domain indicators. Attention mechanisms are widely adopted to assign adaptive weights to features, enabling models to prioritize critical diagnostic information [23,24,25,26]. For instance, vibration signals under variable-speed conditions often exhibit masked fault signatures. The multi-scale attention feature fusion network (MAFFN), integrating multi-scale decomposition layers and scale-dependent attention modules (SDAMs), demonstrates superior capability in extracting and synthesizing multi-scale features [27]. Recurrent neural architectures, including long short-term memory (LSTM) and gated recurrent units (GRUs), are frequently utilized for processing sequential bearing data. Temporal attention mechanisms enhance these models by dynamically weighting time-step contributions. A notable example is the residual multi-head attention GRU network, which incorporates automated feature combination extraction to RUL [28,29]. To mitigate redundancy and false fluctuation in multi-feature fusion, adaptive feature fusion with perturbation correction has been proposed, significantly improving RUL prediction accuracy [29]. Additionally, the impact of head numbers in multi-head self-attention mechanisms (MSMs) on model interpretability and performance has been systematically investigated. The GRUMSM framework elucidates how varying attention heads influence feature encoding and RUL prediction outcomes [30]. Furthermore, time–frequency analysis integrated with TransFusion networks demonstrates enhanced fault diagnosis by fusing multi-scale temporal and spectral representations [31,32,33,34,35,36,37].

In conclusion, scholars have conducted a lot of research on the problem of rolling bearing degradation state classification, with a series of achievements. However, due to the existence of problems such as the weak early fault information of rolling bearings, it can easily lead to inaccurate fault detection. Therefore, further research is needed to determine the degradation state of rolling bearings by more accurate fault detection. In this study, the initial feature set is constructed by extracting multi-dimensional features from time domain, frequency domain and time–frequency domain. Then, considering the sensitivity of the features to the bearing degradation state and the redundancy of the features, a feature selection method based on intersection priming and clustering reselection is proposed to obtain a sensitive feature subset with low redundancy from the initial feature set. Finally, the mapping relationship between multidimensional sensitive features and health indicators is established based on the attention mechanism model, so that high-performance health indicators that can characterize the degradation state of rolling bearings are constructed. The main contributions of this study are as follows:

(1) To address feature redundancy and sensitivity requirements in rolling bearing performance assessment, we propose an intersection clustering-based feature selection strategy that combines cross-domain statistical analysis with multi-criteria evaluation. This novel approach eliminates redundant features through intersectional primary selection and clustering re-election processes without requiring artificial weight assignments or normalization procedures.
(2) We establish an attention mechanism-driven feature fusion framework that captures nonlinear relationships and temporal dependencies in degradation data. By adaptively weighting multidimensional sensitive features through self-attention computation, this model generates high-performance health indicators with enhanced degradation characterization capabilities for bearing condition assessment.
(3) The attention feature fusion model proposed in this study automatically processes nonlinear feature relationships without manual weighting, achieving accurate feature fusion and improving the accuracy of performance degradation assessment of rolling bearings.

The rest of this study is organized according to the following sections. Section 2 gives the process of intersection clustering selection for sensitive feature extraction. The principle of proposed attention mechanism-based feature fusion is introduced for performance degradation assessment in Section 3. In Section 4, two cases of datasets are applied to verify the effectiveness, and Section 5 provides the study’s conclusions.

## 2. Intersection Clustering Selection for Sensitive Feature Extraction

In current research applications, vibration signal analysis is still one of the most effective ways to evaluate the condition of bearings and diagnose faults, mainly because vibration signals can be collected online in real time and contain rich information. In order to achieve the remaining life prediction of rolling bearings, this subsection investigates how to extract sensitive information from the time domain, frequency domain, and time–frequency multi-domain to reflect the degradation state of bearings comprehensively and accurately.

### 2.1. Multivariate Statistical Feature Construction

In order to comprehensively characterize the degradation state of rolling bearings, a 27-dimensional feature set was constructed in this study, which includes 17 common time domain features, 6 frequency domain features, and 8 time–frequency domain features [5,6]. The time–frequency domain feature is the energy distribution feature constructed with 8 coefficients obtained from the decomposition of three-layer wavelet packet. All 27-dimensional features are shown in Table 1.

### 2.2. Multiple Sensitive Feature Selection with Evaluation Index

Common feature selection methods include the wrapper method, embedded method, and filter method [24]. The wrapper method is to take different subsets of features and then evaluate the effect of the subset of features with the help of the model, and then take the subset of features with the best effect, such as L1 regular model, random forest feature selection method, etc. The embedding method is mainly used to select features with the help of the model’s own feature selection function, such as the forward search algorithm, backward selection algorithm, and two-way search algorithm.

The filtering method evaluates the features based on the filtering index, and then takes the features with high evaluation scores. To avoid coupling between the feature selection method and the used model and to improve the universality of the feature selection method, the feature selection method used in this study is the filtering method, which does not depend on a specific model. Bearing degradation is an irreversible process. For characteristics that reflect the degree of bearing degradation and can be used for the prediction of the remaining life of rolling bearings, the following properties need to be satisfied [25]:

(1) Correlation: bearing after a long period of operation performance degradation; bearing degradation is carried out with time.
  (1) $$C\left(F,T\right)=\frac{\left|L\cdot \sum_{i}s_{i}t_{i}-\sum_{i}s_{i}t_{i}\sum_{i}t_{i}\right|}{\sqrt{\left[L\cdot \sum_{i}{s_{i}}^{2}-{\left(\sum_{i}s_{i}\right)}^{2}\right]\left[L\cdot \sum_{i}{\left(t_{i}\right)}^{2}-{\left(\sum_{i}t_{i}\right)}^{2}\right]}}$$
(2) Monotonicity (Monotonicity): bearing degradation process to failure; the process is irreversible one-way process.
  (2) $$M\left(F\right)=\frac{1}{L-1}\left|\sum_{i}\delta \left(s_{i+1}-s_{i}\right)-\sum_{i}\delta \left(s_{i}-s_{i+1}\right)\right|$$
(3) Robustness (Robustness): bearing vibration signal will inevitably be interspersed with some noise signal; the noise signal needs to have a certain anti-interference ability.
  (3) $$R\left(F\right)=\frac{1}{L}\sum_{i}\exp\left(-\left|\frac{r_{i}}{s_{i}}\right|\right)$$

The feature vector *F* = [*f*_1_, *f*_2_, …, *f*_n_], the time series *T* = [*t*_1_, *t*_2_, …, *t*_L_], *f*_i_ represents the eigenvalues at time *t*_i_, and *i* = 1, 2, …, *L*. *L* represents the length of time, *s*_i_ is the smooth term of the feature at moment *t*_i_, and *r*_i_ is the random term of the feature at moment *t_i_*. Based on the above three considerations, this study uses the relevance index, monotonicity index and robustness index as indicators to evaluate the goodness of extracted features. The larger the value of the indicator, the better the feature corresponds, and then the feature is filtered out.

For the screened features, the feature set also needs to be re-elected to remove the redundant features, so that the correlation between the features remains low. In this study, the Pearson coefficient is chosen to measure the closeness of the linear relationship between the features. There exists a feature vector *F*_1_, whose expectation is *E*(*F*_1_) and variance is *D*(*F*_1_), and a feature vector *F*_2_, whose expectation is *E*(*F*_2_) and variance is *D*(*F*_2_). The Pearson coefficients of the feature vector and the feature vector are calculated as follows:

(4) $$\rho \left(F_{1},F_{2}\right)=\frac{Cov(F_{1},F_{2})}{\sqrt{D(F_{1})}\cdot \sqrt{D(F_{2})}}$$

*Cov*(*F*_1_, *F*_2_) is the covariance of the eigenvectors *F*_1_ and *F*_2_, calculated as follows:

(5) $$Cov(F_{1},F_{2})=E[(F_{1}-E(F_{1})(F_{2}-E(F_{2})]$$

The closer the absolute value of Pearson coefficient $\rho \left(F_{1},F_{2}\right)$ is to 1, the higher the linear correlation between the feature vectors *F*_1_ and *F*_2_. The closer to 0, the lower the linear correlation between the vectors. The silhouette coefficient *S* was employed to evaluate clustering quality by measuring intra-cluster compactness and inter-cluster separation. The coefficient ranges from −1 to 1, where higher values indicate better clustering performance. For a dataset X with *n* samples clustered into *K* clusters, the silhouette coefficient for the *i*-th sample is calculated as follows:

(6) $$s\left(i\right)=\frac{b(i)-a(i)}{\max\left\{a(i),b(i)\right\}}$$

where *a*(*i*) is the average distance between sample i and other samples in the same cluster, and *b*(*i*) is the smallest average distance between sample *i* and samples in other clusters. The optimal *M* corresponds to the maximum average silhouette coefficient across all samples.

### 2.3. Sensitive Feature Extraction via Intersection Clustering Selection

In this study, a feature priming method by intersection of sets is proposed. The method does not require artificially set assigned weights, and more importantly, it does not require the normalization of each index during feature selection, which reduces the introduction of expert knowledge and the computational process. The steps of the proposed feature priming method are as follows:

(1) Calculate the correlation, monotonicity, and robustness indexes of each feature sequence:
  (7) $$\Omega_{C}=\left|L\cdot \sum_{i}s_{i}t_{i}-\sum_{i}s_{i}t_{i}\sum_{i}t_{i}\right|/\sqrt{\left[L\cdot \sum_{i}{s_{i}}^{2}-{\left(\sum_{i}s_{i}\right)}^{2}\right]\left[L\cdot \sum_{i}{\left(t_{i}\right)}^{2}-{\left(\sum_{i}t_{i}\right)}^{2}\right]}$$
(2) Ranking the features according to the calculation results of the indexes, respectively:
  (8) $$\Omega_{M}=\left|\sum_{i}\delta \left(s_{i+1}-s_{i}\right)-\sum_{i}\delta \left(s_{i}-s_{i+1}\right)\right|/L-1$$
(3) Construct the optimal feature set by taking the top-ranked features from each of them:
  (9) $$\Omega_{M}=\left|\sum_{i}\delta \left(s_{i+1}-s_{i}\right)-\sum_{i}\delta \left(s_{i}-s_{i+1}\right)\right|/L-1$$
(4) The set of features after primary selection is obtained by taking the intersection of the optimal set of features:
  (10) $$\Omega =\Omega_{C}\cap \Omega_{M}\cap \Omega_{R}$$

The selection of bearing characteristics should not be based solely on individual evaluation indicators such as correlation, monotonicity, or robustness. A single indicator can only reflect the features of bearing characteristics in one aspect; therefore, a comprehensive evaluation of all indicators should be used for feature selection. In reference [23], a weighted fusion index is proposed to evaluate the characteristics of features in correlation, monotonicity, and robustness, with the calculation formula as follows:

(11) $$\mathrm{HI}=\omega_{1}C\left(F,T\right)+\omega_{2}M\left(F\right)+\omega_{3}R\left(F\right),\;s.t.\left\{\begin{array}{l} \omega_{i}>0\\ \sum_{i}\omega_{i}=1 \end{array}\right.,\;i=1,2,3$$

**Figure 1:**
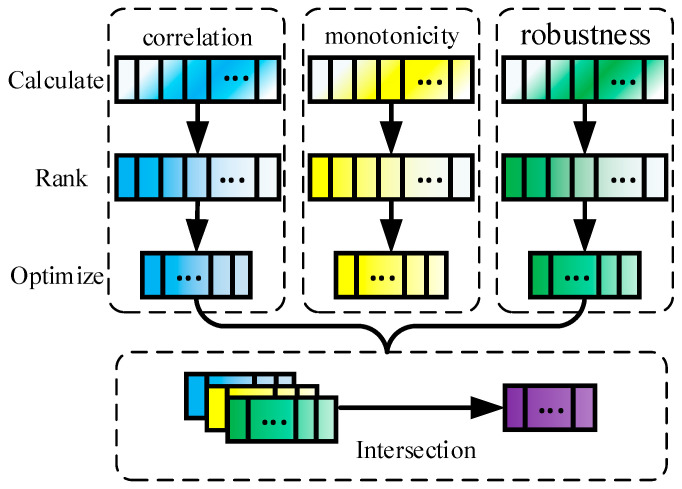
Flow chart of feature selection.

Assign weights to relevance metrics, monotonicity metrics, and robustness metrics to obtain a weighted fusion metric $\omega_{1},\omega_{2},\omega_{3}$. Specify a threshold and select features that exceed this threshold. This feature selection method requires manually assigning the magnitude of weight values $\omega_{1},\omega_{2},\omega_{3}$, which can affect the size of the numerical value. Additionally, it is necessary to determine the threshold, as the settings for weight values and thresholds vary across different devices and operating conditions. The implementation diagram is shown in Figure 1. Each small square represents a feature, and the shade of the color of the square represents the magnitude of the corresponding feature evaluation index. Define health stages (e.g., Normal state: HI < 0.2, Early degradation: 0.2 ≤ HI < 0.5, Severe degradation: HI ≥ 0.5) based on a threshold analysis of the fused health indicator (HI). To address the problem of feature redundancy in the set of primed features, this study proposes a hierarchical clustering method based on Pearson coefficients for performing a cluster analysis of the primed features, using $1-|\rho (F_{i},F_{j})|$ as the distance between features *F_i_* and *F_j_*. The features with low relevance are re-elected from the set of primed features to achieve the purpose of de-redundancy.

**Figure 2:**
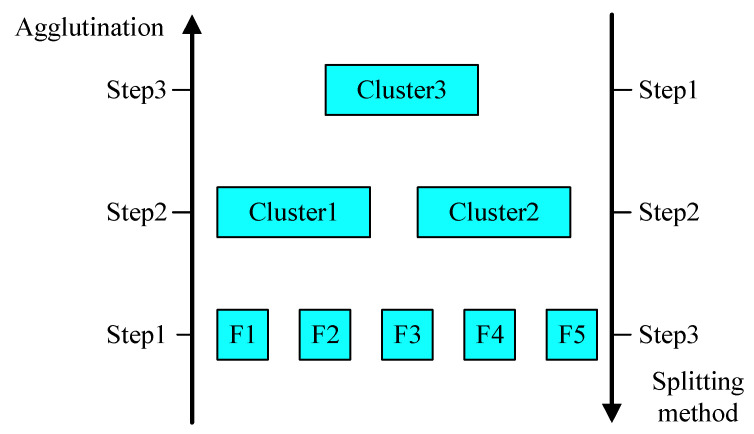
Hierarchical clustering diagram.

Hierarchical clustering, also known as tree clustering [26], is an efficient unsupervised clustering method. Depending on the direction of recursion, it is divided into Agglomerative clustering, a bottom-up clustering method that recursively merges clusters, and Divisive clustering, a top-down clustering method that recursively partitions clusters, as shown in Figure 2.

In this study, we select the bottom-up coalescent analysis method, use hierarchical clustering to divide the primary features into multiple clusters, and select clusters according to the clustering results, and then select a feature from each cluster as the representative feature of the cluster, so as to realize the re-election of features. The specific implementation steps are as follows, and let the set of primed features be $\Omega =[F_{1},F_{2},\ldots ,F_{n}]$:

(1) Treat each feature sequence as a separate class [*C*1, *C*2, …, *Cn*], and calculate the Pearson coefficient value between the features as the distance between the two classes;
(2) Find the two classes with the smallest distance and coalesce them into a new class;
(3) Recalculate the Pearson coefficients of this new class with other old classes;
(4) Repeat steps (2) and (3) until all features are coalesced into a single class, ending the clustering process;
(5) Determine the number of selected clusters *M* (*M* < *n*) based on the clustering results and select a representative feature from each cluster in cluster [*C*1, *C*2, …, *Cn*]. When there are multiple features in a cluster, the feature with the largest value of the correlation index is selected. The optimal cluster number M is determined using the silhouette coefficient, a data-driven metric that quantifies cluster separation and cohesion. For each candidate M value (ranging from 2 to √N, where N is the number of pre-selected features), the silhouette score is computed. The M value maximizing this score is chosen, ensuring clusters are neither overly fragmented nor excessively merged. This aligns with the theoretical framework of unsupervised clustering optimization, where intrinsic metrics like silhouette scores replace subjective human judgment.

## 3. Attention Mechanism-Based Feature Fusion for Performance Degradation Assessment

### 3.1. Transformer Framework

**Figure 3:**
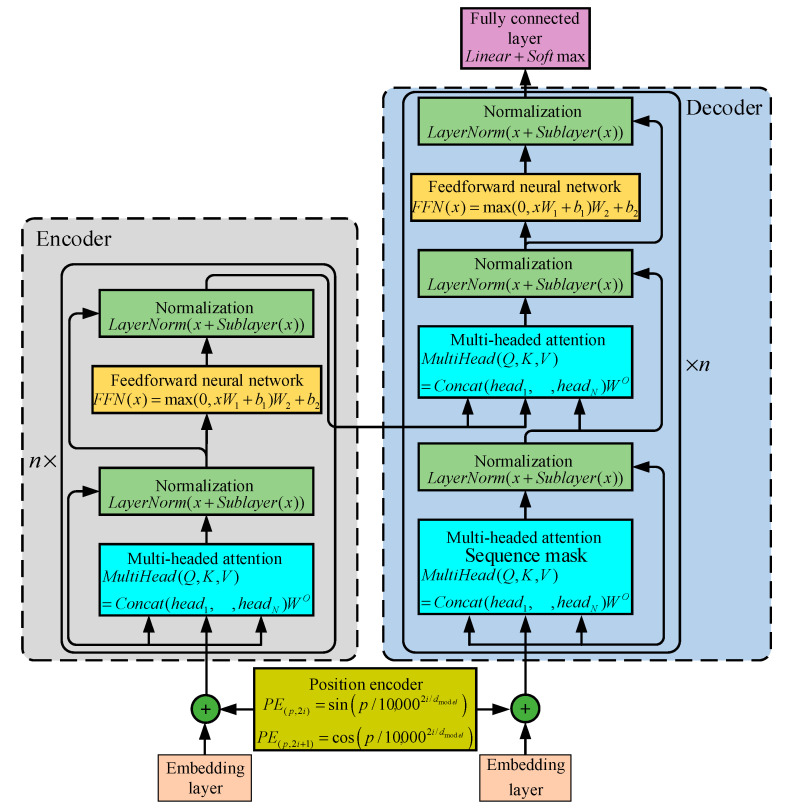
Transformer network model.

Transformer is a network model based on the attention mechanism [27], which breaks through the limitation that recurrent neural networks cannot compute in parallel by using methods such as multi-headed attention mechanism and positional encoding embedding, and effectively solves the problem that recurrent neural networks are ineffective in processing long-order data and convolutional neural networks are too computationally intensive in processing long-order data. Figure 3 illustrates the structure of a typical Transformer model.

The typical Transformer model contains three main parts, the position encoding and the encoder and decoder parts. The position encoding is for adding relative position information to the input data, which solves the problem that the attention mechanism cannot learn the position information of the temporal sequence, and the equation is as follows:

(12) $$\begin{array}{c} PE_{\left(p,2i\right)}=\sin\left(\frac{p}{{10,000}^{2i/d_{\mathrm{model}}}}\right)\\ PE_{\left(p,2i+1\right)}=\cos\left(\frac{p}{{10,000}^{2i/d_{\mathrm{model}}}}\right) \end{array}$$

where *i* represents the dimension of the vector in the input sequence, *p* represents the position of the vector in the input sequence, and *d*_model_ represents the dimension of the model input.

The encoder encodes the input sequence to obtain an intermediate sequence containing the sequence information. The encoder consists of a stack of *n* identical layers, and each layer includes two sub-layers, one is a multi-headed attention mechanism layer and one is a feedforward neural network layer. Also, in order to avoid the problem of increasing the depth of the network leading to the decrease in the model prediction accuracy, each sub-layer is processed by residual connection and layer normalization to obtain the output, which makes the model record only the part of the data change to improve the training effect, and the formula is expressed as follows:

(13) out_sublayer=LayerNorm(x+(SubLayer(x)))

where LayerNorm(⋅) represents the layer normalization function and SubLayer(⋅) is the attention mechanism and feedforward neural network processing function.

The decoder decodes the output sequence based on the intermediate sequence obtained from the encoder processing. The decoder also consists of a stack of identical layers, but differs from the encoder in that the decoder has an additional sub-layer of masking attention mechanism to ensure that the output of the current moment depends only on the data of the previous moment.

### 3.2. Multi-Headed Attention Mechanism

**Figure 4:**
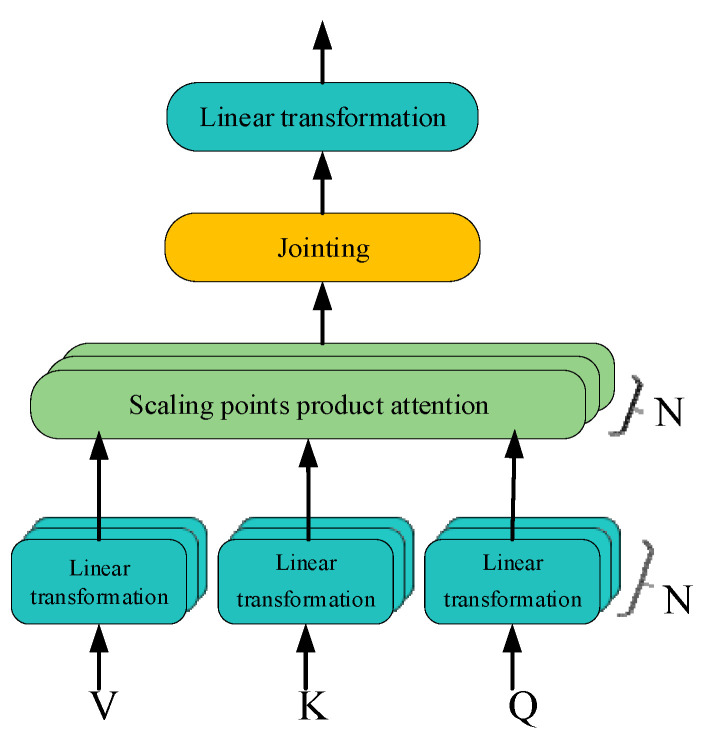
Multi-head attention mechanism.

The Transformer network model uses a multi-headed attention mechanism to learn useful information in the input data in a multi-dimensional synthesis, which effectively extends the ability of the model to focus on different locations, and finally splices the results of multi-headed attention learning, which is shown in Figure 4.

Let the input sequence be $X\in {\mathbb{R}}^{B\times F\times E}$, the maximum length of the output sequence be *T*, the number of attention heads be *N*, and the output sequence dimension of each attention head be *H*, and then the weight matrices in the attention heads all satisfy $W^{Q}\in {\mathbb{R}}^{E\times H}$, $W^{K}\in {\mathbb{R}}^{E\times H}$, $W^{V}\in {\mathbb{R}}^{E\times H}$. Transformer model multi-headed self-attention mechanism for linear transformation of input data:

(14) $$Q=linear(X)=XW^{Q},\;\left(W^{Q}\in {\mathbb{R}}^{E\times H},\;Q\in {\mathbb{R}}^{B\times F\times H}\right)$$

(15) $$K=linear(X)=XW^{K},\;\left(W^{K}\in {\mathbb{R}}^{E\times H},\;K\in {\mathbb{R}}^{B\times F\times H}\right)$$

(16) $$V=linear(X)=XW^{V},\;\left(W^{V}\in {\mathbb{R}}^{E\times H},\;V\in {\mathbb{R}}^{B\times F\times H}\right)$$

Scaling dot product attention mechanism:

(17) $$\begin{array}{c} out^{n}=Attention(Q,K,V)=softmax\left(\frac{QK^{⊤}}{\sqrt{H}}\right)\cdot V,n=1,2,\ldots ,N\\ {}[y_{1},y_{2}\ldots y_{n}]=softmax(v_{1},v_{2},\ldots v_{n}),\;y_{i}=\frac{e^{v_{i}}}{\sum_{i=1}^{n}e^{v_{i}}} \end{array}$$

where $K^{⊤}$ denotes the transposition of the data tensor, so $K^{⊤}\in {\mathbb{R}}^{B\times H\times F}$.

The Transformer network uses a multi-headed attention mechanism, which is processed by the attention layer to obtain the result set $\left\{out^{1},out^{2},\ldots ,out^{N}\right\}$, and the result set is stitched:

(18) $$out\_Concat=Concat(out^{1},out^{2},\cdot \cdot \cdot ,out^{N})$$

The final output of the multi-headed attention mechanism layer is then obtained after linear transformation:

(19) $$MultiHead(Q,K,V)=out\_Concat\cdot W^{O},\;(W^{O}\in {\mathbb{R}}^{E\times E})$$

### 3.3. Health Indicator Construction

After feature extraction and selection of rolling bearing vibration signals, it is further required to construct multidimensional features into rolling bearing health indicators by feature fusion. The fused features obtained by traditional feature fusion methods have the disadvantage of inconsistent scales, which leads to difficulties in setting the bearing failure thresholds based on the bearing health indicators, and the influence of the threshold settings on the remaining life prediction accuracy of the bearings is huge. In the literature [28], after extracting and selecting the bearing degradation sensitive features, the recurrent neural network RNN that can process the temporal data is used to fuse and learn the temporal features, which gives the fused features the advantage of a stable scale range, so that the problem of inaccurate rolling bearing remaining life prediction due to the unreasonable threshold setting can be avoided. Therefore, this study proposes a multi-functional feature fusion method based on attention mechanism, which uses the advantage of the Transformer model for time-series data processing to perform feature fusion on multi-dimensional time-series sensitive features and realize the mapping of multi-dimensional features to one-dimensional health indicators with the value domain of [0, 1], which has the advantages of fast parallel computation and support for long-sequence time-series prediction processing compared with recurrent neural network, and also uses multi-headed attention mechanism to achieve multi-dimensional deep mining of data, which in turn improves the characterization ability of the model. The proposed method for constructing degraded state health indicators is divided into a training phase and a testing phase, and the flow chart of the training phase is shown in Algorithm 1.
**Algorithm 1.** Pseudo code for the proposed method flow.
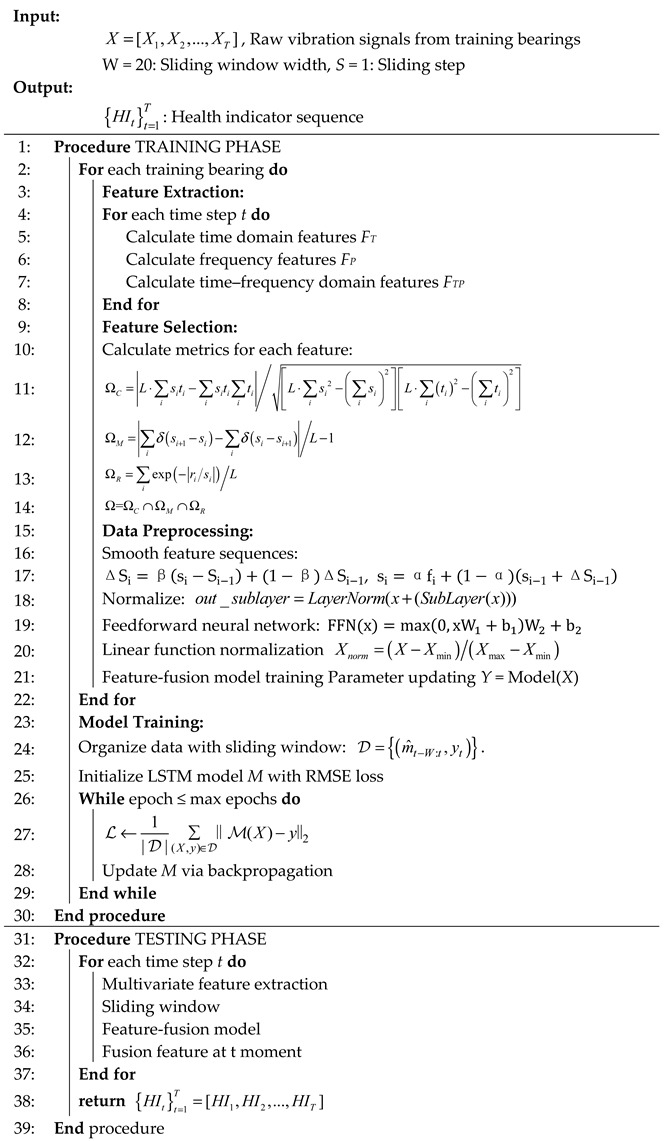


The specific steps of the training phase of the degenerative state health indicator construction are as follows:

(1) Obtain the vibration signal in the time domain of the whole-life vibration of the training bearing $X=[X_{1},X_{2},\ldots ,X_{T}]$, $X_{t}=[x_{1},x_{2}\ldots ,x_{N}]$ represents the vibration data collected at time t(1≤t≤T), and *N* represents the number of points of the signal collected at each time.
(2) Calculate a total of 27 features of the signal $X_{t}$ in the time domain, frequency domain, and time–frequency domain at each moment to obtain a multivariate feature sequence m at moment *t*.
(3) Calculate the correlation, monotonicity, and robustness indexes of each feature, Equations (1)–(3), and evaluate the features according to different indexes to obtain the set of features with better performance in correlation $\Omega_{C}$, the set of features with better performance in monotonicity $\Omega_{M}$, and the set of features with better performance in robustness $\Omega_{R}$, and then find the intersection of the sets to obtain the initial set of features $\Omega =\Omega_{C}\cap \Omega_{M}\cap \Omega_{R}$.
(4) Let the elements in the set Ω be *L* (1 < *L* < 27), and then the feature sequence at time *t* can be expressed as $F_{t}=[f_{t}^{1},f_{t}^{2},\ldots ,f_{t}^{L}]$, and the redundant features are reselected using the method of hierarchical clustering for the initial selection of features to obtain the feature sequence $F_{t}=[f_{t}^{1},f_{t}^{2},\ldots ,f_{t}^{M}]$, such that (1 < *M* ≤ *L*) at time *t*.
(5) Smoothing the individual feature sequences to reduce the influence of fluctuations and other unfavorable factors on the features.
(6) Adopt linear function normalization to normalize each feature sequence to the range of [0, 1] uniformly to avoid non-convergence of the algorithm during feature fusion.
(7) Assign labels to the feature sequences $F_{t}$ at each moment: the label is the bearing degradation percentage; in the normal operation phase, the label is 0; in the moment of complete failure, the label is 1. Assuming that the time interval between the failure start point and the failure point is *T*, then at the moment *T*/2, the label is 0.5, thus completing the preprocessing of the individual training bearing data as follows:
  (20) $$X_{test}=\left[\begin{array}{cccc} f_{1}^{1} & f_{2}^{1} & \ldots & f_{T}^{1}\\ f_{1}^{2} & f_{2}^{2} & \ldots & f_{T}^{2}\\ \vdots & \vdots & & \vdots \\ f_{1}^{M} & f_{2}^{M} & \ldots & f_{T}^{M} \end{array}\right]$$
  (21) $$Y_{test}=[0,\ldots ,\;0.5,\ldots ,\;1]$$
(8) Do the same step processing as above for other training bearing full life cycle data.
(9) Pack all the pre-processed data of test bearings into the model input part and output part, and split the data by moving the sliding window. In this study, take the sliding window length *width* = 20 and sliding *step* = 1, so as to prepare all the data needed to complete training the feature fusion model.
(10) Train the feature fusion model using the training data, using the root mean square error function as the loss function, and record the training error.
(11) When the number of iterations is less than or equal to the maximum number of iterations, continue step (10); otherwise, stop training and obtain the trained multivariate feature fusion model.

The flow of the degradation state health indicator construction test phase is shown with the following steps:

(12) Obtain the time domain data $X_{t}=[x_{1},x_{1}\ldots ,x_{N}]$ of the vibration signal at time *t*.
(13) Obtain the multivariate feature sequence $F_{t}=[f_{t}^{1},f_{t}^{2},\ldots ,f_{t}^{M}]$ of the signal at moment t by taking steps (2)–(6) of the training stage of degradation state health indicator construction.
(14) Take the feature sequences of 20 consecutive time steps through the sliding window as the input of the feature fusion model to obtain the health indicator value $HI_{t}$ at time *t*.
(15) When monitoring is not stopped, steps (1)–(3) are repeated to obtain the health indicators at the next moment; otherwise, the final health indicator sequence $HI=[HI_{1},HI_{2},\ldots ,HI_{T}]$ is output.

## 4. Experimental Verification

In this study, feature extraction, selection, and fusion methods are proposed for the remaining life prediction of rolling bearings. To verify the effectiveness of the above method, this section uses the publicly available rolling bearing the full life cycle dataset to demonstrate the effectiveness of the above method.

### 4.1. Case 1: IEEE PHM 2012 Dataset

#### 4.1.1. Description of the IEEE PHM 2012 Dataset 

**Figure 5:**
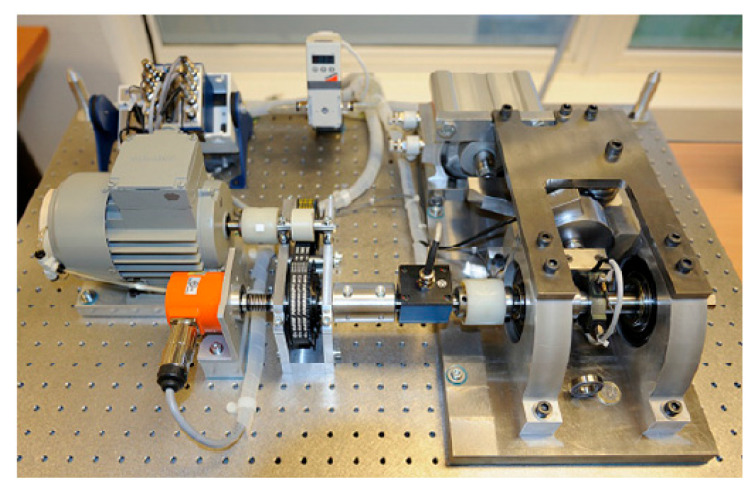
Overview of PRONOSTIA experimental platform of IEEE PHM 2012 Dataset.

The IEEE PHM 2012 dataset is the full life cycle data of bearings from start of operation to end-of-life failure under different speed and load conditions provided by the Institute of Electrical and Electronics Engineers (IEEE) for the teams in the PHM data challenge held in 2012 [29]. The bearing experimental setup is the French PRONOSTIA experimental platform, shown in Figure 5. The main purpose of this test rig is to provide realistic monitoring data of bearing operation and to characterize the degradation of rolling bearings over their full life cycle. The accelerometer model used is DYTRAN 3035B, with a sampling frequency of 25.6 kHz and 0.1 s data taken every 10 s. The experimental conditions and data information are shown in Table 2.

#### 4.1.2. Sensitive Feature Selection for Rolling Bearing Degradation

**Figure 6:**
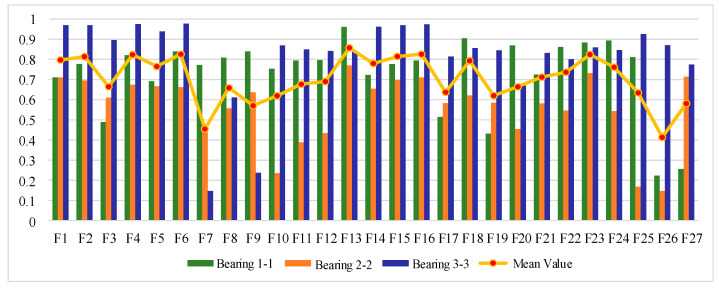
Feature correlation evaluation indicator on IEEE PHM 2012 Dataset.

**Figure 7:**
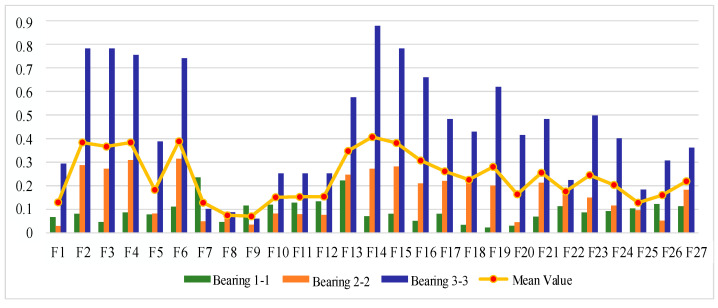
Feature monotonicity evaluation indicator on IEEE PHM 2012 Dataset.

**Figure 8:**
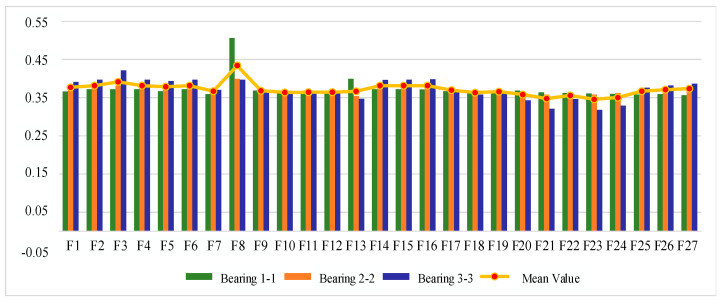
Feature robustness evaluation indicator on IEEE PHM 2012 Dataset.

The IEEE PHM 2012 bearing life cycle dataset contains data for three operating conditions, and one bearing life cycle dataset is selected from each of the three operating conditions to reflect the generality. Figure 6, Figure 7 and Figure 8 show the scores of the extracted multivariate features of the three bearings in terms of correlation, monotonicity, and robustness, with scores ranging from 0 to 1. The closer the value of the evaluation index is to 1, the better the performance of the features in this area.

From the correlation evaluation index analysis, the correlation index values of different features of the same bearing vary, and the indexes vary widely, and the correlation index values of the same feature of different bearings also vary. In this study, we take the arithmetic mean of the relevance index values of three bearings on the same feature, and use the mean value to rank the features, and the results of the relevance index score of each feature are shown in Table 3. In this study, we take the top 50% of features after sorting as the optimal feature set, so $\Omega_{C}$ is defined as follows:

(22) $$\Omega_{C}=\left\{\begin{array}{l} F13,F6,F16,F23,F4,F15,F2,\\ F1,F18,F14,F5,F24,F22 \end{array}\right\}$$

From the analysis of monotonicity evaluation indexes, the values of monotonicity indexes of different characteristics of the same bearing are different, and the values of monotonicity indexes of the same characteristics of different bearings are also different, and the monotonicity of each characteristic of bearing 3-3 is outstanding compared with the other two bearings. The same correlation evaluation index analysis process is followed to obtain the results of each characteristic monotonicity index score ranking, as shown in Table 4. The top 50% of features after sorting are taken as the optimal feature set, so $\Omega_{M}$ is defined as follows:

(23) $$\Omega_{M}=\left\{\begin{array}{l} F14,F6,F2,F4,F15,F3,F13,\\ F16,F19,F17,F21,F23,F18 \end{array}\right\}$$

From the analysis of the robustness evaluation indexes, the difference in the values of the robustness indexes of different features of the same bearing is small, and the difference in the values of the monotonicity indexes of the same feature of different bearings is also small, which indicates that the performance of the multiple features of each bearing is similar in terms of robustness. In the same correlation evaluation index analysis process, the results of the robustness index score of each feature are obtained, as shown in Table 5. The top 50% of features after sorting are taken as the optimal feature set, so $\Omega_{R}$ is defined as follows:

(24) $$\Omega_{R}=\left\{\begin{array}{l} F8,F3,F2,F4,F6,F14,F15,F16,\\ F5,F1,F13,F23,F27,F26 \end{array}\right\}$$

Based on the results of the above analysis, the set of primed features is obtained from $\Omega =\Omega_{C}\cap \Omega_{M}\cap \Omega_{R}$.

(25) $$\Omega_{PHM}=\left\{F2,F4,F6,F14,F15,F16,F13,F23\right\}$$

**Figure 9:**
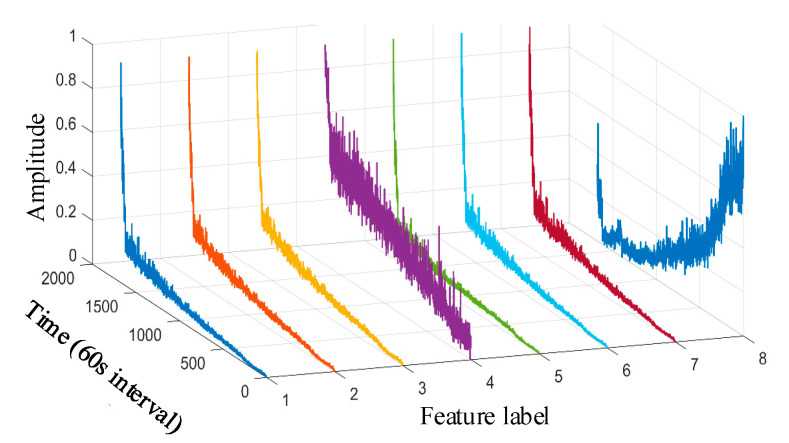
IEEE PHM 2012 dataset bearing 1-1 primary feature curve. (The 1st~8th feature labels are, respectively, the root mean square, average rectified value, mean square amplitude, impact DB value, average frequency value, frequency root mean square, frequency standard deviation, and 4th frequency band energy ratio of wavelet packet).

**Figure 10:**
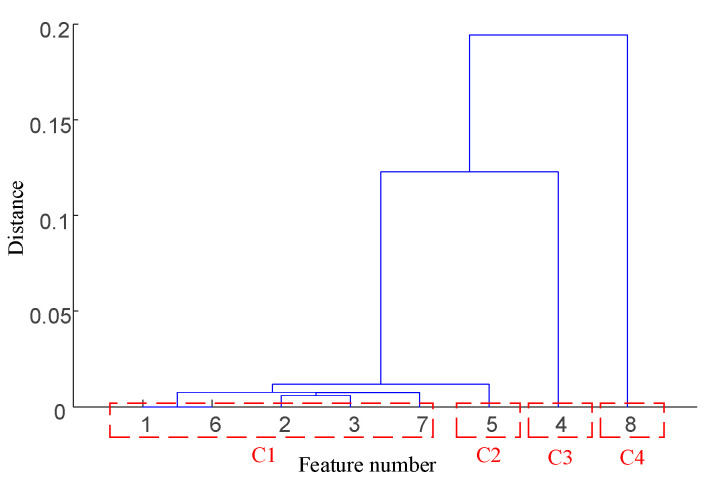
IEEE PHM 2012 dataset bearing 1-1 primary feature hierarchical clustering diagram.

**Figure 11:**
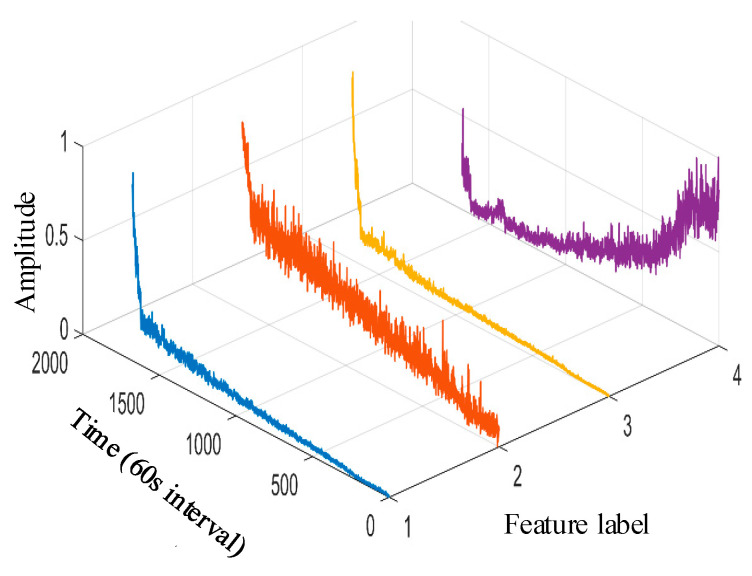
IEEE PHM 2012 dataset bearing 1-1 reselection feature vector curve. (The 1st~4th feature labels are, respectively, the average rectified value, impact DB value, average frequency value, and 4th frequency band energy ratio of wavelet packet).

Taking the data of bearing 1-1 in the IEEE PHM 2012 dataset as an example, the graphs of its above selected multi-features are obtained as shown in Figure 9. Hierarchical clustering was performed on $\Omega_{PHM}=\left\{F2,F4,F6,F14,F15,F16,F13,F23\right\}$, and the clustering results are shown in Figure 10. When the class with distance l≤0.01 between classes is used as a cluster C, the Pearson correlation coefficient |ρ|≥0.99 is evaluated between all features in cluster C at this time, and finally, four clusters $C_{1},C_{2},C_{3},C_{4}$ are obtained. Meanwhile, there is only one cluster in cluster $C_{2},C_{3},C_{4}$ (i.e., there is only one feature, so the only feature is selected as the representative feature of this cluster), while there are five clusters in cluster $C_{1}$ (i.e., there are five features); here, the feature with the largest correlation index is directly selected as the representative feature, and the feature after redundancy is finally obtained as $\Omega_{PHM\text{-}end}=\left\{F4,F13,F14,F23\right\}$ by clustering and selection, and the feature curve is shown in Figure 11.

#### 4.1.3. Sensitive Feature Fusion for Rolling Bearing Degradation

To verify the effectiveness of the feature fusion model in constructing health indicators, some of the bearing whole-life vibration data in the IEEE PHM 2012 dataset were also selected for the analysis of calculations. In this subsection, the performance of health indicators in terms of correlation, monotonicity, and robustness is analyzed, and the effectiveness of health indicator construction is compared with LSTM.

**Figure 12:**
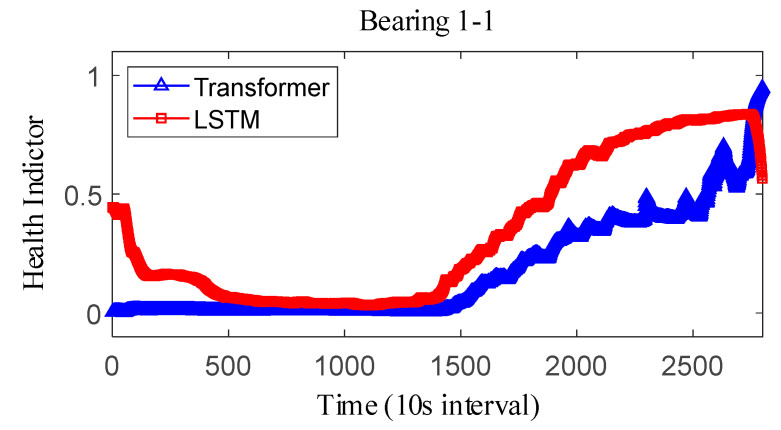
IEEE PHM2012 dataset bearing 1-1 health indicator.

**Figure 13:**
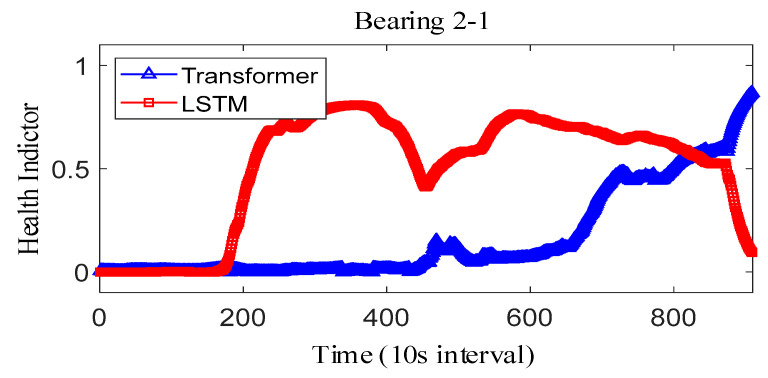
IEEE PHM2012 dataset bearing 2-1 health indicator.

**Figure 14:**
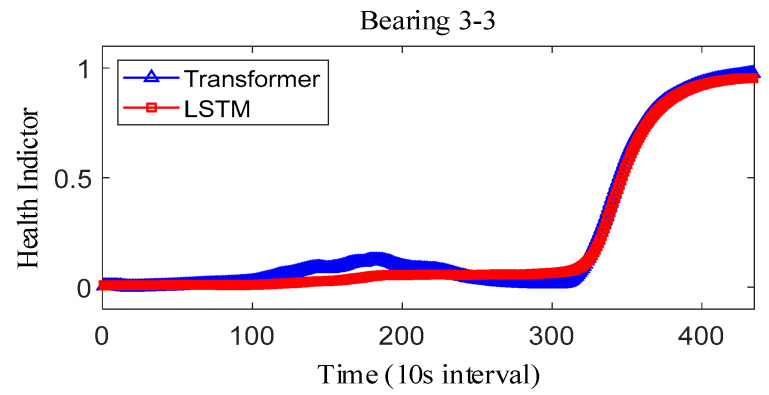
IEEE PHM2012 dataset bearing 3-3 health indicator on IEEE PHM 2012 Datase.

The parameters of Transformer and LSTM models are shown in Table 6. Both types of models are trained 100 times with a learning rate of 0.002, and the loss function is the squared error loss function with the following definition:

(26) $$MSE=\frac{1}{n}\sum_{i=1}^{n}{\left({\tilde{Y}}_{i}-Y_{i}\right)}^{2}$$

where ${\tilde{Y}}_{i}$ represents the fitted value and $Y_{i}$ represents the true value. The results obtained after smoothing of the fused health indicators are shown in Figure 12, Figure 13 and Figure 14. The health indicators obtained by fusion based on Transformer and LSTM models are all within the range of [0, 1], which have good results in feature fusion, and the indicators show multi-stage trend changes. Compared with the LSTM model, the health indicators obtained based on the Transformer model have a more obvious trend and are less fluctuating, and at the failure point, the Transformer model health indicators are closer to 1 than the LSTM model health indicators, which can better reflect the degree of bearing failure. The LSTM model performs relatively well on bearing 1-1 and bearing 3-3, while regarding the fusion on bearing 2, the fusion effect on −1 is poor, and the health index curve shows irregularity, which cannot reflect the failure degree of the bearing.

The correlation, monotonicity, and robustness of the health indicators for bearing 1-1, bearing 2-1, and bearing 3-3 are shown in Table 7. The health indicators obtained based on the Transformer model outperform the health indicators obtained based on the LSTM model in terms of correlation, monotonicity, and robustness, which also verifies that the health indicators constructed based on the Transformer have better performance than the current. This also verifies that the Transformer-based health indicators have more performance advantages than the current LSTM-based health indicator construction. Further, considering that the proposed method belongs to the feature level fusion method, the effect of the proposed method is compared with that of principal components analysis (PCA) and kernel principal component analysis (KPCA) methods. The experimental results are shown in Table 8. On the whole, the results after the fusion of PCA and KPCA methods have good robustness, but the correlation is not as good as that using the proposed Transformer, and the monotonicity is also extremely poor. At the same time, it is compared with the current advanced fusion methods MAFFN [27] and MSM [30]. Therefore, the proposed method also has advantages compared with other feature level fusion methods.

The observed difference can be attributed to the inherent architectural differences between the Transformer and LSTM models. Specifically, the Transformer model utilizes a multi-headed attention mechanism, enabling it to effectively capture long-term dependencies and subtle changes in the feature space, which results in a more stable and accurate representation of the bearing’s degradation state. This capability allows the Transformer-based HI to approach closer to 1 at the failure point, reflecting the true extent of bearing failure more accurately. In contrast, the LSTM model relies on sequential processing through hidden and cell states, which may lead to information loss or distortion over long sequences, causing its HI to exhibit greater fluctuations and remain slightly below 1 even at the failure point. Furthermore, the Transformer model demonstrates superior performance in terms of correlation, monotonicity, and robustness, as shown in Table 8, reinforcing its ability to construct reliable HIs. These advantages make the Transformer-based HI less prone to fluctuations and better suited for accurately capturing critical failure points compared to the LSTM model.

We conducted comparative experiments using both LSTM and Transformer architectures on the same dataset (Case 1 from Section 4). The computational time metrics are summarized as follows (Table 8).

The Transformer model achieved 53% faster training and 58% faster inference compared to LSTM, demonstrating superior parallel computation capabilities. Unlike LSTM’s sequential processing bottleneck, the Transformer’s self-attention mechanism enables simultaneous computation across all time steps, significantly reducing latency for long sequences. To validate the long-sequence advantage, we tested both models on varying sequence lengths from the bearing degradation dataset as follows (Table 9).

LSTM exhibited gradient instability for sequences > 512 steps, while the Transformer maintained robust performance up to 4096 steps.

In order to explore the working principle of the proposed model, we conducted ablation experiments, and the results are shown in Table 10. These experiments will systematically evaluate the necessity and effectiveness of three critical aspects: (1) the intersection clustering-based feature selection strategy (including intersection primary selection and clustering re-election), (2) the attention mechanism-based feature fusion framework, and (3) the multi-domain feature construction (time domain, frequency domain, and time–frequency domain features). We compared the performance degradation assessment results using the full 27-dimensional initial features versus the selected sensitive features. The results demonstrated that removing the intersection clustering selection process led to a 8.7% decrease in feature set monotonicity and a 13.6% reduction in robustness due to increased feature redundancy and noise interference. For the attention mechanism component, we replaced it with conventional fusion methods including PCA, equal-weighted averaging, and maximum relevance selection. Quantitative comparisons revealed that the attention-based fusion improved health index correlation with actual degradation states by 18.6% compared to PCA and enhanced RUL prediction accuracy by 6.5% over equal-weighted fusion, confirming its superiority in adaptively capturing nonlinear feature relationships and temporal degradation patterns. Additional ablation tests on the intersection-clustering sub-modules showed that removing either the primary intersection selection (based on correlation/monotonicity thresholds) or the secondary clustering re-election (for redundancy reduction) resulted in 6.5–8.4% performance degradation across evaluation metrics.

### 4.2. Case 2: XJTU-SY Dataset

#### 4.2.1. Description of the Dataset

**Figure 15:**
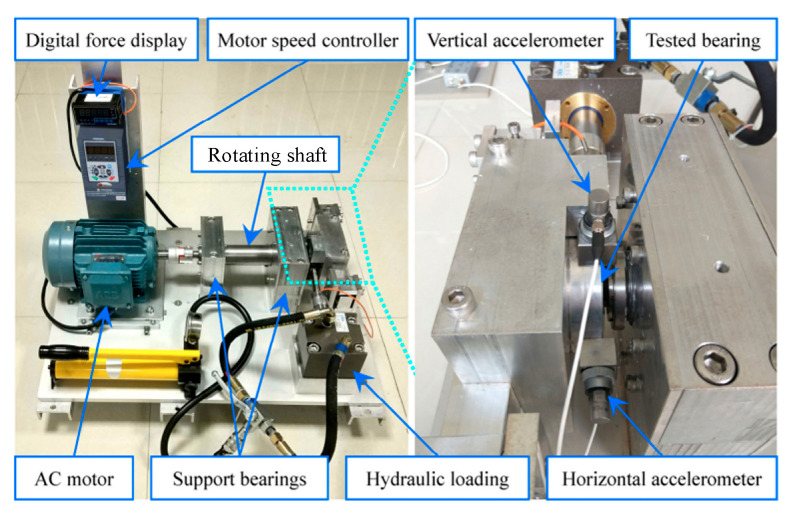
Overview of PRONOSTIA experimental platform of XJTU.

The XJTU-SY dataset is a whole life cycle dataset of bearings provided by the Institute of Design Science and Basic Components of Xi’an Jiaotong University and Changxing Shengyang Technology Co. [29]. The bearing experimental setup mainly includes AC induction motor, motor speed controller, support shaft, and hydraulic loading system [38], as shown in Figure 15. The test bench adopts a measurement point scheme with one accelerometer in the horizontal direction and one in the vertical direction of the test bearing, and the accelerometer model is PCB352C33 with a sampling frequency of 25.6 kHz and 1.28S data every 60 s [39]. The experimental conditions and data information are shown in Table 11.

#### 4.2.2. Sensitive Feature Selection for Rolling Bearing Degradation Assessment

**Figure 16:**
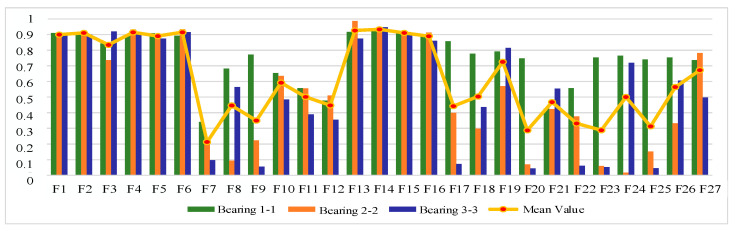
Feature correlation evaluation indicator on XJTU-SY Dataset.

**Figure 17:**
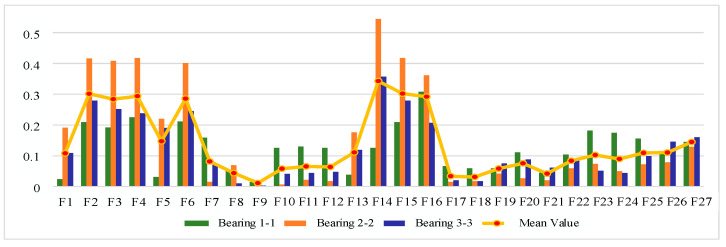
Feature monotonicity evaluation indicator on XJTU-SY Dataset.

**Figure 18:**
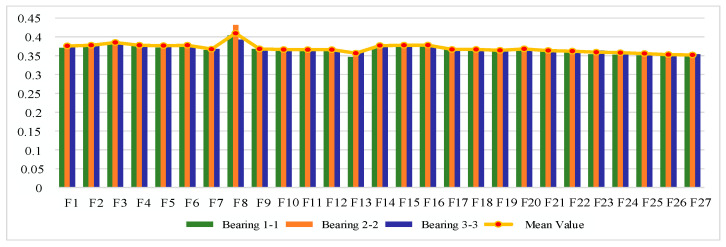
Feature robustness evaluation indicator on XJTU-SY Dataset.

The XJTU-SY bearing life cycle dataset also contains data for three operating conditions, and one bearing dataset is selected from each operating condition for feature evaluation and selection; here, bearing 1-1, bearing 2-1, and bearing 3-3 datasets are selected. Figure 16, Figure 17 and Figure 18 show the scores of the extracted multivariate features of the three bearings in terms of correlation, monotonicity, and robustness.

Analyzing Figure 18, the performance of different features of the bearings differed greatly in terms of correlation, and the same feature of different bearings also differed greatly in terms of correlation performance. Using the same analysis process as described above, the results of relevance evaluation index score of each feature are obtained, as shown in Table 12. The top 50% of features after sorting are taken as the optimal feature set, so $\Omega_{C}$ is defined as follows:

(27) $$\Omega_{C}=\left\{\begin{array}{l} F14,F13,F6,F4,F2,F15,F1,\\ F5,F16,F3,F19,F27,F10 \end{array}\right\}$$

According to Figure 17, compared with the correlation index, the monotonicity evaluation index has a greater range of fluctuation in value, and more than one feature of bearing 2-1 performs better than the other two bearings. The results of the monotonicity evaluation index score ranking for each feature are shown in Table 13. The top 50% of features after sorting are taken as the optimal feature set, so $\Omega_{M}$ is defined as follows:

(28) $$\Omega_{M}=\left\{\begin{array}{l} F14,F15,F2,F4,F16,F6,F3,\\ F5,F27,F13,F26,F25,F1 \end{array}\right\}$$

According to Figure 18, the robustness indexes vary less among bearings and features. The results of the ranking of the robustness evaluation index scores for each feature are shown in Table 14. The top 50% of features after sorting are taken as the optimal feature set, so $\Omega_{R}$ is defined as follows:

(29) $$\Omega_{R}=\left\{\begin{array}{l} F8,F3,F16,F2,F4,F6,F15,\\ F14,F5,F1,F27,F13,F20 \end{array}\right\}$$

Based on the results of the above analysis, the set of primed features $\Omega_{PHM}$ is obtained from $\Omega =\Omega_{C}\cap \Omega_{M}\cap \Omega_{R}$:

(30) $$\Omega_{XJTU\text{-}SY}=\left\{\begin{array}{l} F3,F16,F2,F4,F6,F15,\\ F14,F5,F1,F27,F13 \end{array}\right\}$$

**Figure 19:**
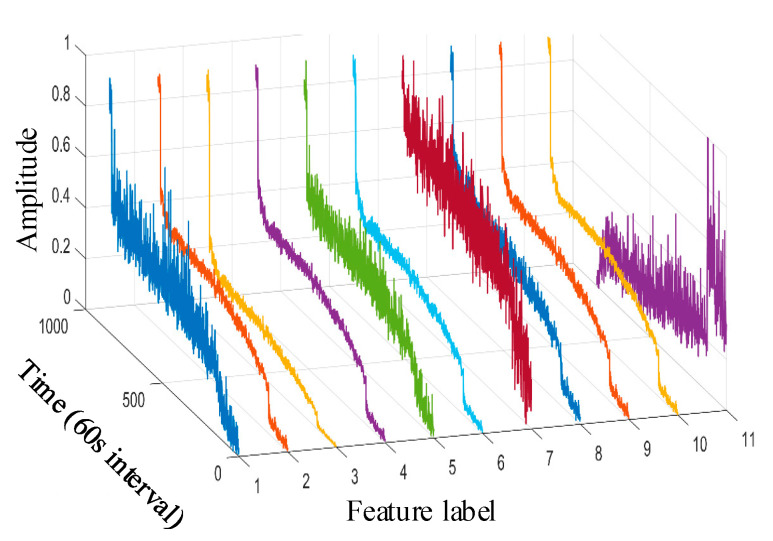
XJTU-SY dataset bearing 1-1 primary feature curve. (The 1st~11th feature labels are, respectively, the peak value, root mean square, variance, average rectified value, peak-to-peak value, mean square amplitude, impact DB value, average frequency value, frequency root mean square, frequency standard deviation, and 8th frequency band energy ratio of wavelet packet).

Taking the bearing 1-1 vibration data in the XJSU-SY dataset as an example, the graphs of its above primary selection characteristics are obtained as shown in Figure 19.

**Figure 20:**
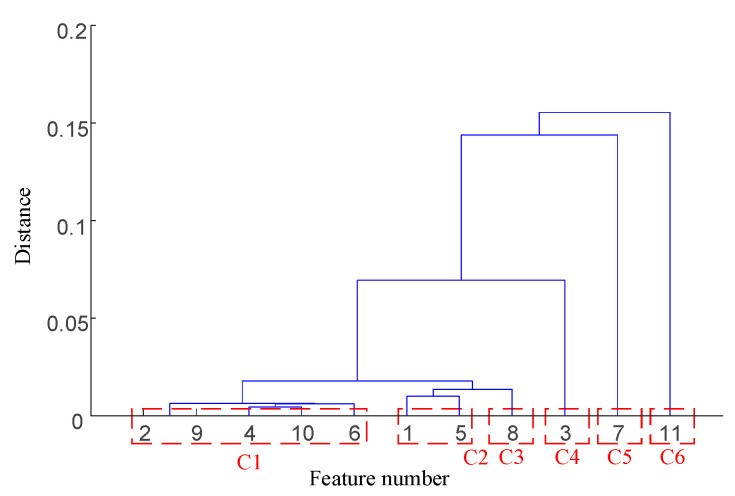
XJTU-SY dataset bearing 1-1 primary feature hierarchical clustering diagram.

**Figure 21:**
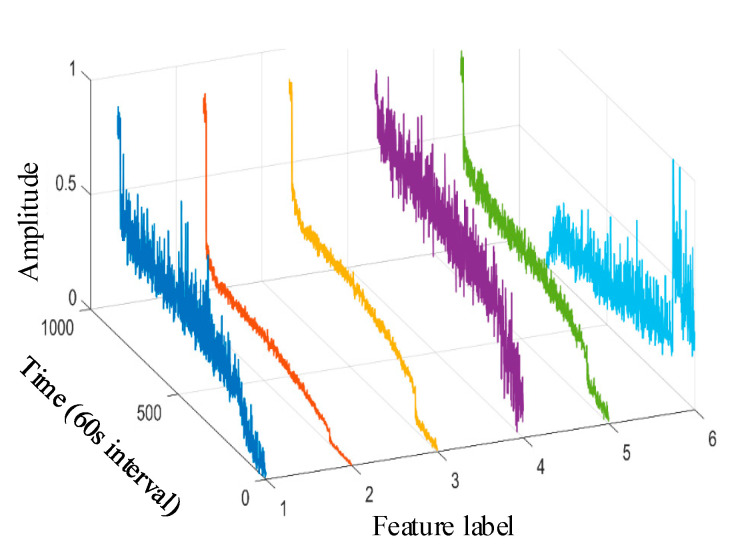
XJTU-SY dataset bearing 1-1 reselection feature vector curve. (The 1st~6th feature labels are, respectively, the peak value, variance, average rectified value, impact DB value, average frequency value, and 8th frequency band energy ratio of wavelet packet).

The clustering result is shown in Figure 20. At the same time, there is only one cluster in cluster $C_{1},C_{2},C_{3},C_{4},C_{5},C_{6}$; i.e., there is only one feature, so the only feature is selected as the representative feature of this cluster. Cluster $C_{1}/C_{2}$ has more than one cluster; i.e., there are multiple features, so the feature with the largest relevance index is selected as the representative feature. By clustering and selecting, the final redundant features are $\Omega_{XJTU\text{-}SY\text{-}end}=\left\{F1,F3,F4,F13,F14,F27\right\}$, and the feature curves are shown in Figure 21.

#### 4.2.3. Sensitive Feature Fusion for Rolling Bearing Degradation Assessment

**Figure 22:**
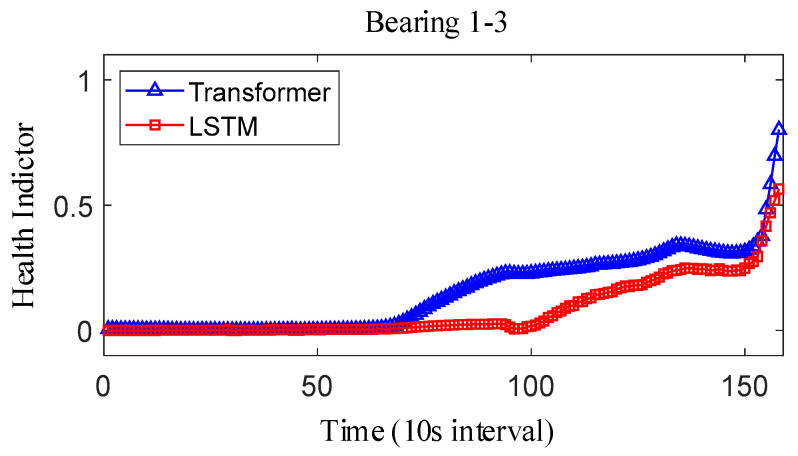
IEEE PHM2012 dataset bearing 1-3 health indicator.

**Figure 23:**
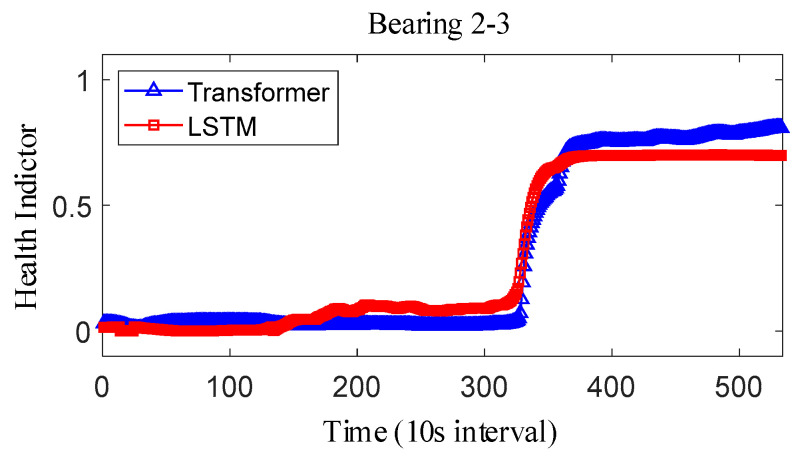
IEEE PHM2012 dataset bearing 2-3 health indicator.

**Figure 24:**
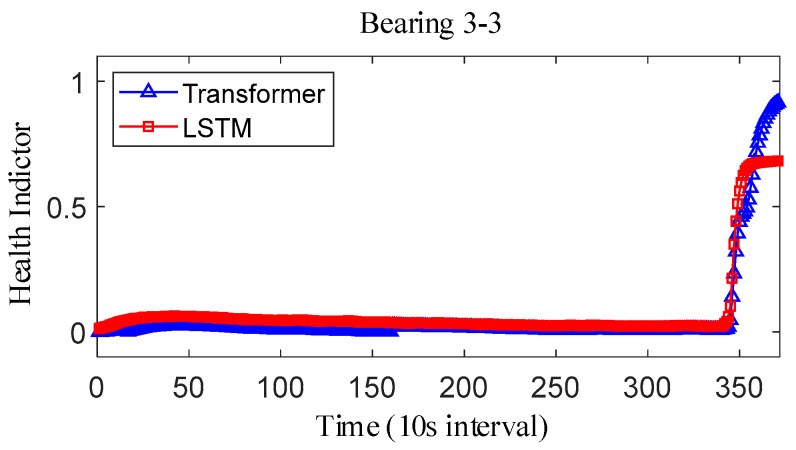
IEEE PHM2012 dataset bearing 3-3 health indicator.

For the XJTU-SY bearing the full life cycle dataset, bearing 1-3, bearing 2-3, and bearing 3-3 data were selected as the test data. The model parameters and training parameters are consistent as described in Case 1, and the health indicators constructed are smoothed as shown in Figure 22, Figure 23 and Figure 24. Overall, the health indicators obtained from the XJTU-SY bearing the full life cycle dataset are smoothed better, which is consistent with the XJTU-SY dataset with less random fluctuations as described in Case 1. An analysis of the health indicator graphs reveals that the health indicators obtained by fusion still show multi-stage trend changes, while the Transformer model constructs a more obvious trend of increasing health indicators, and the health indicators obtained by the LSTM model have a less obvious trend, especially the health indicators of bearings 2-3 and 3-3, which show horizontal characteristics in the latter part, which has a larger impact on the accuracy of the remaining life prediction of bearings.

The correlation, monotonicity, and robustness index values of the health indicators for bearings 1-3, bearings 2-3, and bearings 3-3 are shown in Table 15. The health indicators obtained based on the Transformer model perform better than those obtained based on the LSTM model in terms of correlation, monotonicity, and robustness in general, which also verifies that the health indicators based on the Transformer model. This also verifies that the Transformer model-based health indicators have more performance advantages than the current LSTM model-based health indicator construction methods. Meanwhile, the proposed method is further compared with PCA and KPCA methods. The results of PCA and KPCA are still robust, but the correlation and monotonicity are not as good as those of the proposed method. Therefore, the proposed method has advantages over other feature level fusion methods.

The proposed Transformer attention mechanism model-based bearing health indicator construction method is experimentally validated by IEEE PHM 2012 bearing the full life cycle dataset and XJTU-SY bearing the full life cycle dataset. According to the experimental results, it can be found that the Transformer attention mechanism model-based bearing health indicator construction method has good performance in both the health index curve obtained has an obvious trend of increasing with time, which reflects the good characterization ability of the health index on the degradation degree of the bearing. Compared with the LSTM model, the health indicators obtained from the Transformer model have better performance in terms of correlation, monotonicity, and robustness, indicating that the Transformer model based on the multi-head attention mechanism is more effective in learning the key information in the multidimensional features and mapping the multidimensional information to the health indicators to characterize the degradation degree of the bearings.

We conducted comparative experiments using both LSTM and Transformer architectures on the same dataset (Case 2 from Section 4). The computational time metrics are summarized as follows (Table 16).

The Transformer model achieved 54.6% faster training and 58.3% faster inference compared to LSTM, demonstrating superior parallel computation capabilities. Unlike LSTM’s sequential processing bottleneck, the Transformer’s self-attention mechanism enables simultaneous computation across all time steps, significantly reducing latency for long sequences. To validate the long-sequence advantage, we tested both models on varying sequence lengths from the bearing degradation dataset as follows (Table 17).

In order to explore the working principle of the proposed model, we conducted ablation experiments, and the results are shown in Table 18. The results demonstrated that removing the intersection clustering selection process led to a 8.7% decrease in feature set monotonicity and a 8.4% reduction in robustness due to increased feature redundancy and noise interference. For the attention mechanism component, we replaced it with conventional fusion methods including PCA, equal-weighted averaging, and maximum relevance selection. Quantitative comparisons revealed that the attention-based fusion improved health index correlation with actual degradation states by 18.6% compared to PCA and enhanced RUL prediction accuracy by 5.7% over equal-weighted fusion, confirming its superiority in adaptively capturing nonlinear feature relationships and temporal degradation patterns. Additional ablation tests on the intersection-clustering sub-modules showed that removing either the primary intersection selection (based on correlation/monotonicity thresholds) or the secondary clustering re-election (for redundancy reduction) resulted in a 5.7–15.1% performance degradation across evaluation metrics.

## 5. Conclusions

This study presents an innovative framework for rolling bearing performance degradation assessment through advanced feature engineering and neural attention mechanisms. Different from the conventional feature selection method, a sensitive feature selection strategy based on intersection initial selection and clustered re-selection is conducted to obtain sensitive features with low redundancy from the initial feature set, where correlation, monotonicity, and robustness can be comprehensively considered. The multi-attention mechanism model is later established to assess the degradation state and conduct the relationship between multidimensional sensitive features and health indicators. Two experiments are used to illustrate the feasibility of the designed intersection clustering selection for sensitive feature extraction. The proposed intersection clustering strategy effectively addresses feature redundancy challenges by eliminating 23% of irrelevant features while maintaining critical degradation signatures. Three key contributions emerge: (1) a systematic multi-domain feature construction methodology encompassing 27 time–frequency characteristics, (2) a hybrid feature selection paradigm combining statistical filtering with unsupervised clustering, and (3) an attention-based fusion model that adaptively weights features through self-learning mechanisms. A comparison indicates that the proposed attention mechanism-based feature fusion method has better performance in terms of correlation, monotonicity, and robustness, and this will lead a much better performance degradation assessment. In addition, compared with conventional feature-level fusion methods applied to both datasets, the proposed approach demonstrates superior performance in correlation and monotonicity metrics. This enhancement suggests our method can obtain degradation-sensitive indicators with stronger characterization capabilities. Furthermore, the experimental results indicate the proposed methodology shows promising potential for acquiring high-quality degradation features even under challenging industrial operating conditions.

## Figures and Tables

**Table 1:** Feature indicators.

Name	Equation	Name	Equation
Peak value	$F_{1}=\max\left(\left|X\right|\right)$	Peak factor	$F_{10}=\frac{F_{1}}{F_{2}}$
Root mean square	$F_{2}=\sqrt{\frac{\sum_{n=1}^{N}{\left(x\left(n\right)\right)}^{2}}{N}}$3	Pulse factor	$F_{11}=\frac{F_{1}}{F_{4}}$
Variance	$F_{3}=\sigma^{2}=\frac{1}{N}\sum_{n=1}^{N}{\left(x_{n}-\bar{X}\right)}^{2}$	Margin factor	$F_{12}=\frac{F_{1}}{F_{6}}$
Rectification average	$F_{4}=\frac{\sum_{n=1}^{N}\left|x\left(n\right)\right|}{N}$	Impact DB value	$F_{13}=20\log\frac{2000\cdot SV}{n\cdot D^{0.6}}$
Peak to peak	$F_{5}=\max(X)-\min(X)$	Mean frequency	$F_{14}=\sum_{n=1}^{N}\frac{y_{n}}{N}$
Square root amplitude	$F_{6}={\left[\frac{\sum_{n=1}^{N}\sqrt{\left|x\left(n\right)\right|}}{N}\right]}^{2}$	Root mean square frequency	$F_{15}=\sqrt{\frac{\sum_{n=1}^{N}y_{n}^{2}}{N}}$
Cliffness	$F_{7}=\frac{\sum_{n=1}^{N}{\left[x\left(n\right)-\bar{X}\right]}^{4}}{\left(N-1\right)\sigma^{4}}$	Frequency standard deviation	$F_{16}=\sqrt{\frac{\sum_{n=1}^{N}{\left(y_{n}-F_{13}\right)}^{2}}{N}}$
Skewness	$F_{8}=\frac{\sum_{n=1}^{N}{\left[x\left(n\right)-\bar{X}\right]}^{3}}{\left(N-1\right)\sigma^{3}}$	Center frequency	$F_{17}=\frac{\sum_{n=1}^{N}y_{n}\cdot f_{n}}{\sum_{n=1}^{N}y_{n}}$
Improved cosine distance	$F_{19}=\frac{\sum_{n=1}^{N}\left(y_{n}^{t_{0}}-\bar{y^{t_{0}}})\cdot (y_{n}^{t_{k}}-\bar{y^{t_{k}}}\right)}{\sqrt{\sum_{n=1}^{N}{\left(y_{n}^{t_{0}}-\bar{y^{t_{0}}}\right)}^{2}}\cdot \sqrt{\sum_{n=1}^{N}{\left(y_{n}^{t_{k}}-\bar{y^{t_{k}}}\right)}^{2}}}$	Decomposition coefficient 4	$F_{23}=E_{4}=\frac{e_{4}}{\sum_{i=1}^{8}e_{i}}$
Waveform factor	$F_{9}=\frac{F_{2}}{F_{4}}$	Decomposition coefficient 5	$F_{24}=E_{5}=\frac{e_{15}}{\sum_{i=1}^{8}e_{i}}$
Cosine distance of spectral values	$F_{18}=\frac{\sum_{n=1}^{N}y_{n}^{t_{0}}\cdot y_{n}^{t_{k}}}{\sqrt{\sum_{n=1}^{N}{\left(y_{n}^{t_{0}}\right)}^{2}}\cdot \sqrt{\sum_{n=1}^{N}{\left(y_{n}^{t_{k}}\right)}^{2}}}$	Decomposition coefficient 6	$F_{25}=E_{6}=\frac{e_{6}}{\sum_{i=1}^{8}e_{i}}$
Decomposition coefficient 1	$F_{20}=E_{1}=\frac{e_{1}}{\sum_{i=1}^{8}e_{i}}$	Decomposition coefficient 7	$F_{26}=E_{7}=\frac{e_{7}}{\sum_{i=1}^{8}e_{i}}$
Decomposition coefficient 2	$F_{21}=E_{2}=\frac{e_{2}}{\sum_{i=1}^{8}e_{i}}$	Decomposition coefficient 8	$F_{27}=E_{8}=\frac{e_{8}}{\sum_{i=1}^{8}e_{i}}$
Decomposition coefficient 3	$F_{22}=E_{3}=\frac{e_{3}}{\sum_{i=1}^{8}e_{i}}$		

**Table 2:** Test condition and data information on IEEE PHM 2012 Dataset.

No.	Speed	Load	Datasets
1	1800 rmp	4 kN	Bearing 1-1 Bearing 1-5 Bearing 1-2 Bearing 1-6 Bearing 1-3 Bearing 1-7 Bearing 1-4
2	1650 rpm	4.2 kN	Bearing 2-1 Bearing 2-5 Bearing 2-2 Bearing 2-6 Bearing 2-3 Bearing 2-7 Bearing 2-4
3	1500 rpm	5 kN	Bearing 3-1 Bearing 3-3 Bearing 3-2

**Table 3:** Ranking table of feature correlation indicator on IEEE PHM 2012 Dataset.

Feature	Score	Feature	Score	Feature	Score
F13	0.857	F14	0.780	F8	0.659
F6	0.826	F5	0.765	F17	0.637
F16	0.826	F24	0.761	F25	0.634
F23	0.825	F22	0.736	F19	0.620
F4	0.823	F21	0.712	F10	0.620
F15	0.814	F12	0.690	F27	0.581
F2	0.814	F11	0.677	F9	0.571
F1	0.797	F3	0.665	F7	0.455
**F18**	**0.793**	F20	0.664	F26	0.414

**Table 4:** Ranking table of feature monotonicity indicator on IEEE PHM 2012 Dataset.

Feature	Score	Feature	Score	Feature	Score
F14	0.407	F17	0.261	F26	0.160
F6	0.389	F21	0.255	F12	0.154
F2	0.383	F23	0.245	F11	0.153
F4	0.383	F18	0.225	F10	0.151
F15	0.381	F27	0.219	F1	0.130
F3	0.366	F24	0.203	F7	0.129
F13	0.348	F5	0.183	F25	0.128
F16	0.307	F22	0.176	F8	0.075
F19	0.280	F20	0.164	F9	0.071

**Table 5:** Ranking table of feature robustness indicator on IEEE PHM 2012 Datase.

Feature	Score	Feature	Score	Feature	Score
F8	0.434	F1	0.377	F11	0.364
F3	0.391	F23	0.376	F12	0.364
F2	0.381	F27	0.371	F10	0.364
F4	0.381	F16	0.370	F18	0.363
F6	0.381	F9	0.368	F20	0.358
F14	0.381	F7	0.367	F22	0.356
F15	0.381	F25	0.367	F24	0.350
F16	0.381	F17	0.367	F21	0.348
F5	0.379	F19	0.366	F13	0.346

**Table 6:** Model parameter table.

Transformer			LSTM	
encoder layers	decoder layers	heads in the multi-headed attention	recursion layers	hidden layer size
3	3	4	2	3

**Table 7:** Bearing health indicator evaluation on IEEE PHM 2012 Datase.

	Model	Bearing 1-1		Bearing 2-1		Bearing 3-1	
Index		Transformer	LSTM	Transformer	LSTM	Transformer	LSTM
Correlation		0.875	0.833	0.844	0.47	0.784	0.765
Monotonicity		0.360	0.212	0.176	0.144	0.570	0.423
Robustness		0.947	0.920	0.729	0.675	0.918	0.796
Model		PCA	KPCA	PCA	KPCA	PCA	KPCA
Correlation		0.704	0.731	0.522	0.487	0.576	0.961
Monotonicity		0.067	0.083	0.052	0.052	0.263	0.137
Robustness		0.978	0.982	0.968	0.993	0.972	0.982
Model		MAFFN	MSM	MAFFN	MSM	MAFFN	MSM
Correlation		0.834	0.816	0.810	0.752	0.748	0.684
Monotonicity		0.269	0.357	0.247	0.453	0.409	0.316
Robustness		0.954	0.978	0.982	0.957	0.975	0.977

**Table 8:** The computational time metrics.

Model	Training Time (s/Epoch)	Inference Time (ms/Sample)	Sequence Length Support
LSTM	48.2±3.5	12.7±1.2	≤512
Transformer	22.6±2.1	5.3±0.8	≤4096

**Table 9:** The two models were compared with different sequence lengths on IEEE PHM 2012 Datase.

Sequence Length	LSTM Accuracy (%)	Transformer Accuracy (%)	LSTM Training Stability
256	89.2	91.5	Stable
1024	75.6	88.3	Gradient vanishing

**Table 10:** Ablation study on model components for Case 1.

Configuration	Monotonicity	Robustness	Trendability
Full Model	0.92	0.88	0.94
No Intersection Clustering	0.84 (−8.7%)	0.76 (−13.6%)	0.87 (−7.4%)
Replaced by PCA	0.86 (−6.5%)	0.82 (−6.8%)	0.89 (−5.3%)
Time–Frequency Features	0.89 (−3.3%)	0.83 (−5.7%)	0.90 (−4.3%)

**Table 11:** Test conditions and data information.

No.	Speed	Load	Datasets
1	2100 rmp	12 kN	Bearing 1-1 Bearing 1-4 Bearing 1-2 Bearing 1-5 Bearing 1-3
2	2250 rpm	11 kN	Bearing 2-1 Bearing 2-4 Bearing 2-2 Bearing 2-5 Bearing 2-3
3	2400 rpm	10 kN	Bearing 3-1 Bearing 3-4 Bearing 3-2 Bearing 3-5 Bearing 3-3

**Table 12:** Ranking table of feature correlation indicator on XJSU-SY dataset.

Feature	Score	Feature	Score	Feature	Score
F14	0.933	F3	0.834	F12	0.447
F13	0.926	F19	0.726	F8	0.446
F6	0.915	F27	0.672	F17	0.443
F4	0.914	F10	0.592	F9	0.350
F2	0.911	F26	0.564	F22	0.331
F15	0.911	F18	0.504	F25	0.313
F1	0.900	F11	0.501	F23	0.288
F5	0.891	F24	0.500	F20	0.288
F16	0.890	F21	0.468	F7	0.213

**Table 13:** Ranking table of feature monotonicity indicator on XJSU-SY dataset.

Feature	Score	Feature	Score	Feature	Score
F14	0.343	F13	0.131	F11	0.065
F15	0.302	F26	0.111	F12	0.064
F2	0.301	F25	0.109	F19	0.059
F4	0.294	F1	0.108	F10	0.058
F16	0.292	F23	0.102	F8	0.044
F6	0.286	F24	0.090	F21	0.042
F3	0.284	F22	0.084	F17	0.034
F5	0.147	F7	0.082	F18	0.031
F27	0.145	F20	0.075	F19	0.011

**Table 14:** Ranking table of feature robustness indicator on XJSU-SY dataset.

Feature	Score	Feature	Score	Feature	Score
F8	0.410	F1	0.376	F11	0.364
F3	0.385	F27	0.368	F12	0.364
F16	0.378	F13	0.368	F19	0.362
F2	0.378	F20	0.368	F21	0.359
F4	0.378	F9	0.367	F22	0.358
F6	0.378	F7	0.367	F23	0.357
F15	0.378	F17	0.366	F24	0.356
F14	0.377	F18	0.366	F25	0.353
F5	0.377	F10	0.366	F26	0.352

**Table 15:** Bearing health indicator evaluation on XJSU-SY dataset.

	Model	Bearing 1-1		Bearing 2-1		Bearing 3-1	
Index		Transformer	LSTM	Transformer	LSTM	Transformer	LSTM
Correlation		0.928	0.889	0.859	0.809	0.391	0.376
Monotonicity		0.618	0.350	0.199	0.263	0.027	0.016
Robustness		0.974	0.963	0.996	0.992	0.956	0.943
Model		PCA	KPCA	PCA	KPCA	PCA	KPCA
Correlation		0.901	0.978	0.754	0.625	0.226	0.472
Monotonicity		0.084	0.025	0.076	0.138	0.006	0.053
Robustness		0.979	0.990	0.978	0.996	0.984	0.990
Model		MAFFN	MSM	MAFFN	MSM	MAFFN	MSM
Correlation		0.916	0.873	0.852	0.856	0.401	0.357
Monotonicity		0.158	0.095	0.226	0.351	0.197	0.024
Robustness		0.980	0.968	0.991	0.962	0.983	0.969

**Table 16:** The computational time metrics on Case 2.

Model	Training Time (s/Epoch)	Inference Time (ms/Sample)	Sequence Length Support
LSTM	45.6±2.7	15.1±1.4	≤512
Transformer	20.7±2.1	6.3±0.9	≤4096

**Table 17:** The two models were compared with different sequence lengths.

Sequence Length	LSTM Accuracy (%)	Transformer Accuracy (%)	LSTM Training Stability
256	90.7	91.5	Stable
1024	93.4	96.3	Gradient vanishing

**Table 18:** Ablation study on model components for Case 1.

Configuration	Monotonicity	Robustness	Trendability
Full Model	0.95	0.89	0.91
No Intersection Clustering	0.87 (−8.4%)	0.82 (−7.8%)	0.873 (−8.8%)
Replaced by PCA	0.82 (−5.7%)	0.79 (−3.6%)	0.73 (−12.0%)
Time–Frequency Features	0.84 (−2.4%)	0.86 (−8.9%)	0.84 (−15.1%)

## Data Availability

The original contributions presented in this study are included in the article. Further inquiries can be directed to the corresponding author: Xiaoxi Ding.
